# Neighborhood poverty and hopelessness in older adults: The mediating role of perceived neighborhood disorder

**DOI:** 10.1371/journal.pone.0311894

**Published:** 2024-10-15

**Authors:** Yeon Jin Choi, Eun Young Choi, Jennifer A. Ailshire

**Affiliations:** 1 College of Social Work, University of Kentucky, Lexington, Kentucky, United States of America; 2 Leonard Davis School of Gerontology, University of Southern California, Los Angeles, California, United States of America; PLoS ONE, UNITED STATES OF AMERICA

## Abstract

Hopelessness is one of the strongest predictors of health and mortality, particularly for older populations. Prior research has found associations between individual-level socioeconomic factors and hopelessness, but less is known about the potential importance of neighborhood-level socioeconomic contexts for hopelessness. In particular, the role of neighborhood disorder as a potential explanatory factor for poor psychological well-being remains underexplored. This study investigates whether neighborhood poverty is associated with a sense of hopelessness among older adults and if perceived neighborhood disorder mediates the link between poverty and hopelessness. Individual-level data came from the 2014/2016 Health and Retirement Study and were merged with neighborhood-level poverty data from the 2012–2016 and 2014–2018 American Community Survey. Linear regression models were employed to examine the association between neighborhood poverty, disorder, and hopelessness. Respondents in neighborhoods with higher poverty levels reported a greater sense of hopelessness (b = 0.11, 95% CI = 0.08, 0.15, *p* < .001), controlling for individual-level sociodemographic and health characteristics. Greater perceived neighborhood disorder was also positively associated with a sense of hopelessness (b = 0.16, 95%CI = 0.14, 0.18). When we included both neighborhood poverty and disorder in the same model, the association between neighborhood poverty and hopelessness was reduced by two thirds (b = 0.04, 95%CI = 0.0003, 0.07), while the association between perceived disorder and hopelessness remained robust (b = 0.16, 95%CI = 0.14, 0.18). We further examined the formal mediating effects of neighborhood disorder using structural equation modeling. The total effect of neighborhood poverty on hopelessness was significant (β = 0.08, bootstrapped 95%CI = 0.05, 0.10). The direct effect of neighborhood poverty was not significant (β = 0.02, bootstrapped 95% CI = -0.01, 0.04), while the indirect effect through neighborhood disorder was significant (β = 0.06, bootstrapped 95% CI = 0.05, 0.07). Neighborhood disorder mediated 75% of the association between neighborhood poverty and hopelessness. In light of these findings, improving neighborhood conditions, such as signs of disorder, may alleviate feelings of hopelessness in older adults residing in impoverished neighborhoods.

## Introduction

### Background

Hopelessness refers to a negative expectation about the future, coupled with a sense of personal futility and loss of motivation [1]. The risk of hopelessness is notably high in older age, partly because aging-associated challenges, such as reduced mobility and independence from declines in physical functioning or chronic health conditions, financial strain from reduced income or increased medical expenses, and social isolation from loss of close relationships and social support [2, 3]. These challenges may make older adults feel they lack abilities and resources to adjust to the changes that accompany the aging process, thereby diminishing motivation and undermining a positive outlook toward the future. According to Hamzaoglu and colleagues [4], 56% of adults aged 65 and above experienced feelings of hopelessness, a rate nearly three times higher than the 18% observed among those aged 15–24. The impact of hopelessness extends across various health and well-being outcomes in later life. Empirical evidence suggests that middle-aged and older adults who feel hopeless are at an elevated risk for cognitive impairment [5], cardiovascular disease [6], loneliness [7], depression [8], and mortality [9]. Furthermore, hopelessness has emerged as a strong predictor of suicidal ideation [10, 11] and a pronounced unwillingness to receive life-sustaining medical interventions [12] among older adults. Given its importance for health and well-being and its differential impact on older adults, identifying factors to reduce hopelessness in the older population represents a public health priority.

The learned hopelessness theory [13] offers a useful theoretical foundation for identifying potential risk factors. It posits that consistent exposure to uncontrollable and adverse environmental conditions gradually fosters a belief that such adverse situations are inescapable, culminating in a sense of helplessness [13]. Prior research has primarily focused on individual-level socioeconomic disadvantage and shown a strong association between educational attainment [14] and income [15] with a subjective sense of failure and subsequent hopelessness. However, little attention has been given to neighborhood socioeconomic disadvantage despite its potentially crucial role in the development of sense of hopelessness.

### Neighborhood poverty and hopelessness

Neighborhoods can serve as a key upstream source, influencing the degree of stressors and access to opportunities [16]. Impoverished neighborhoods are often exposed to more chronic stressors, such as unemployment, violence, and crime [17], and offer fewer resources, including limited access to healthcare, opportunities for physical activity, and social interactions, than affluent neighborhoods [18–20]. In prior research, neighborhood poverty has been found to be associated with worse psychological well-being, including greater levels of depression and loneliness [20–22]. Similarly, area-level concentration of economic and social challenges can lower residents’ hope for future opportunities. In a sample of young adults aged 18–24 in greater Seattle area, Snedker & Hooven [23] explored the role of neighborhood stressors in psychological well-being and found that perceived neighborhood poverty was associated with an increased sense of hopelessness. While this first and only study on neighborhood poverty and hopelessness provides initial evidence suggesting the potential of neighborhood poverty to shape one’s negative perceptions of the future, it is based on a young adult sample in a highly selected geographic area, and therefore the role of neighborhood poverty in hopelessness in older adulthood remains unclear. The effects of neighborhood context are likely to be accentuated among older adults due to age-related functional limitations, mobility decline, and reduction in social networks that can lead to greater reliance on immediate residential environments [24]. Thus, we expect that greater neighborhood poverty will be associated with a greater sense of hopelessness among older adults (**Hypothesis 1**).

### Neighborhood disorder as a mediator

Neighborhoods with higher levels of poverty may be harmful for psychological well-being due to the presence of stressors with known relationships to health. Social disorganization theory [25] provides a framework for conceptualizing the underlying process through which structural factors of neighborhoods, such as poverty rates, shape the larger social context. This theory posits that structural disadvantages, exemplified by high poverty rates, create a stressful social environment by disrupting the development and maintenance of social controls and community ties that are essential for addressing communal challenges [25]. Impoverished areas often lack resources and investments for maintenance, repair, and other essential services needed to uphold public order and social control [26, 27], which consequently contribute to shifting social norms toward non-conventional behaviors and incivilities. Such disruptions result in social decline and community decay, which is operationalized as neighborhood disorder. Neighborhood disorder is defined as “the physical and social features of neighborhoods that may signal the breakdown of order and social control (p. 4325)” [28], and is represented by features such as litter, graffiti, vandalism, noise, rundown and poorly maintained buildings and dwellings, drug and alcohol use, and conflicts among neighbors [26, 29, 30]. Empirical studies substantiate this theory, demonstrating that impoverished neighborhoods exhibit reduced social control and heightened physical disorder [29–31]. This degradation of neighborhood social conditions, in turn, can exert downstream effects on residents’ health and well-being [32–34]. Previous research has reported the detrimental impact of neighborhood disorder on poor health and psychological well-being outcomes of older adults, including anxiety, depression, cognition, and physical functioning [35–38]. Moreover, Ross and Mirowsky [26] emphasized the cascading psychological effects of neighborhood disorder, highlighting how its prevalence heightens fear while diminishing the perception of control over one’s life, fostering a sense of powerlessness to escape adverse circumstances. Empirical studies have provided evidence that neighborhood disorder acts as a mediator through which neighborhood disadvantage operates on residents’ psychological well-being. For instance, both Ross [26] and Kim [39] found that the association between neighborhood disadvantage–an index created based on the prevalence of poverty and mother-only households–and depression was largely explained by perceived neighborhood disorder. Based on the theoretical and empirical evidence, we anticipate that neighborhood disorder would mediate the association between neighborhood poverty and a sense of hopelessness (**Hypothesis 2**).

### Present study

This study leverages data from a nationally representative sample of middle-aged and older adults to examine the association between neighborhood poverty and hopelessness. Furthermore, we conduct mediation analysis to assess neighborhood disorder as a potential intermediate pathway linking neighborhood poverty to hopelessness. Elucidating the underlying mechanisms of this association can contribute to a better understanding of how neighborhood socioeconomic disadvantage influences feelings of hopelessness, but also may guide intervention strategies. If the relationship between neighborhood poverty and hopelessness is mediated by increased disorder, targeted interventions to reduce such disorder may mitigate the risk of hopelessness.

## Materials and methods

### Data

Individual-level data came from the Health and Retirement Study (HRS), a nationally representative, longitudinal survey of US adults aged 50 years and older. HRS respondents are asked about a wide range of information such as sociodemographic factors, health status, and neighborhood characteristics. In 2006, a random half of the HRS sample was administered the Psychological and Lifestyle Self-Administrated Questionnaire (SAQ), which includes items measuring sense of hopelessness and perceived neighborhood disorder, with the other half completing the SAQ in 2008 [40]. The most recent SAQ panel was 2018/2020, which includes respondents surveyed during the pandemic, a time when older adults may have felt hopeless for reasons related to the global pandemic. Therefore, we used the next most recent data from the 2014/2016 SAQ. There were 6,972 age-eligible respondents in 2014 and 6,102 in 2016 who completed and returned the SAQ, respectively. Among the respondents who completed the SAQ in 2014 or 2016 (N = 13,074), we excluded 618 (4.7%) who have missing information on the following individual-level variables (missing may overlap): perceived neighborhood disorder (*n* = 296), hopelessness (*n* = 292), education (*n* = 54), race/ethnicity (*n* = 44), and marital status (*n* = 9), and activity of daily limitations (*n* = 1).

Data on neighborhood poverty come from the U.S. Census Bureau’s American Community Survey (ACS) tract-level 5-year estimates data, which have been linked to HRS respondent information and are available from the HRS Contextual Data Resource (HRS-CDR) [41]. HRS geocodes respondent addresses and uses that information to determine the census tract in which they reside. We linked respondents from the 2014 HRS survey to 2012–2016 ACS poverty data, and the 2016 HRS respondents to 2014–2018 poverty data. We excluded respondents who did not have valid census tract identifiers (*n* = 162) or neighborhood poverty data (*n* = 10). The final analytical sample consisted of 12,284 adults aged 50 years and older.

### Measures

#### Hopelessness

Hopelessness was measured with four items used in prior work [1, 42]: “I feel it is impossible for me to reach the goals that I would like to strive for;” “The future seems hopeless to me and I can’t believe that things are changing for the better,” “I don’t expect to get what I really want;” and “There is no use in really trying to get something I want because I probably won’t get it.” Respondents rated each question on a six-point Likert scale (1 = *strongly disagree*; 6 = *strongly agree*). We averaged across item responses to create a summary score of hopelessness, treating any cases with two or more missing items as missing on the summary score [40]. A higher mean score represented a greater sense of hopelessness (range: 1 to 6; α = 0.85 for the 2010 data, α = 0.88 for the 2012 data, α = 0.88 for the 2014 data, and α = 0.87 for the 2016 data).

#### Neighborhood poverty

In the ACS, a person’s poverty status is determined using a set of dollar value thresholds that differ by family size and composition. If one’s total family income in the past 12 months is less than the appropriate threshold for that family type, then they are considered to be in poverty. We created a measure of neighborhood poverty for each census tract by dividing the population with poverty-level income by the total population for whom poverty status was determined (possible range: 0 to 1). We created a log-transformed variable of neighborhood poverty to correct for skewness, in line with prior research [43]. A higher value in this logged variable indicates a neighborhood experiencing greater poverty.

#### Perceived neighborhood disorder

Perceived neighborhood disorder was assessed using respondent reports about the following four aspects of their neighborhoods, defined as the “area within a 20-minute walk or about a mile” from the respondent’s home: “vandalism/graffiti are a big problem in this area,” “this area is full of rubbish,” “there are many vacant/deserted houses,” and “people would be afraid to walk alone in this area after dark” [44]. Respondents rated each item on a seven-point Likert-type scale to indicate the extent to which respondents agree with each statement (1 = *least agree*; 7 = *most strongly agree*). An average disorder score was created by averaging across responses to the four items (range: 1 to 7), with higher scores representing higher levels of neighborhood disorder. Respondents who had two or more missing items were coded as missing [40]. The Cronbach’s alpha was .84 in 2014 and .83 in 2016.

#### Covariates

We selected demographic, socioeconomic, and health covariates a priori, guided by previous research demonstrating their close associations with both neighborhood conditions and hopelessness, serving as potential confounders [14, 15, 27, 45]. Individual-level variables included age, sex, race/ethnicity (0 = non-Hispanic white, 1 = non-Hispanic Black, 2 = Hispanic, or 3 = non-Hispanic other), marital status (0 = married/partnered, 1 = divorced/separated, 2 = widowed, or 3 = never married), educational attainment (in years), total household income (logged), and limitations in activities of daily living (ADLs) including bathing, dressing, walking across a room, and getting in and out of bed (0 = no limitations or 1 = any limitations). Additionally, in the sensitivity analyses, we also controlled for prior values of hopelessness in our models (measured in 2010/2012) to mitigate the potential for earlier levels of hopelessness to influence subsequent perceptions of neighborhood disorder and to account for individual differences in the propensity to feel hopelessness.

### Analytical strategy

We estimated a series of linear regression models to examine the association between neighborhood poverty and a sense of hopelessness. In the initial model, we assessed the association of neighborhood poverty with hopelessness, adjusting for individual-level sociodemographic characteristics. In the second model, we examined the link between perceived neighborhood disorder and hopelessness, The final model included both neighborhood poverty and perceived disorder to determine whether perceived disorder could explain the association between neighborhood poverty and hopelessness observed in the first model.

To further quantify the mediating role of perceived neighborhood disorder, we conducted a formal mediation analysis using structural equation modeling (SEM). SEM estimates all model parameters simultaneously, producing more accurate standard errors for the path coefficients compared to the regression technique [46, 47]. We first conducted a confirmatory factor analysis to validate the measurement model for our latent variables–perceived neighborhood disorder and hopelessness. Subsequently, in the structural model, we performed the effect decomposition. Here, the direct effect was represented as the path from poverty to hopelessness while controlling for neighborhood disorder and other covariates. The indirect effect was the path from poverty to hopelessness through neighborhood disorder, independent of other covariates. The total effect was the sum of the direct and indirect effects. Individual-level confounders were included as predictors of neighborhood disorder and hopelessness. The overall fit of the model was evaluated using the following criteria: a root mean square error of approximation (RMSEA) below 0.05, a comparative fit index (CFI) above 0.95, and a standardized root mean square residual (SRMR) under 0.08 [48, 49]. We assessed mediated effects based on 95% bootstrapped confidence intervals (1,000 iterations). Effect sizes were quantified in proportion to the total effects mediated by neighborhood disorder [50].

All analyses incorporated sampling weights to account for nonresponse and differential probabilities of selection and to make the sample representative of the U.S. population. We used single-level regression models given minimal clustering at the census tract level in our sample, where the average number of respondents per tract was only 2.2. A significant portion of the sample lived in tracts with few individuals (22% in a singleton tract, 24% in tracts with two people, 9% in tracts with three people, and only 17% in tracts with more than ten individuals). Analyses using the multilevel models produced largely similar estimates to the single level models. Nevertheless, we adjusted the standard errors for within-cluster correlation to mitigate the risk of underestimating standard errors in single-level models. Regression analyses were performed using Stata 17.0 [51], and mediation analyses were computed with Mplus 8.0 [52].

### Ethical approval

We used data from the Health and Retirement Study (HRS), which is sponsored by the National Institute on Aging (NIA U01AG009740) and conducted by the University of Michigan following written informed consent under a protocol approved by an IRB at the institution. While most data are publicly available, access to study participants’ geographic location and the HRS-CDR used for this study is regulated and requires a formal application process due to privacy and confidentiality requirements. Approval for this study was obtained from the Institutional Review Board (UP-13-00397-CR006) at the University of Southern California.

## Results

Table 1 presents the sample characteristics. The average hopelessness score was 2.25 (SD = 1.27) on a 6-point scale, indicating relatively low levels of hopelessness among our sample. The average neighborhood poverty rate in our sample was 14% (Mean proportion = 0.14; SD = 0.11). The mean score of perceived neighborhood disorder was 2.48 (SD = 1.41) on a 7-point scale, suggesting lower levels of disorder overall. The sample comprised a majority of women (53.5%), non-Hispanic white (76.7%), and married/partnered (66.2%). The mean age was 66.2 (SD = 9.93), with a mean educational attainment of 13.4 years (SD = 2.92), and an average total household income of $89,792. 14.7% of the sample reported having one or more limitations in daily activities of living.

**Table 1:** Sample characteristics, 2014/2016 health and retirement study, age 50+ (N = 12,284).

	Full Sample
	M(SD)/%
Hopelessness (range: 1–6) ^a^	2.25 (1.27)
Neighborhood poverty (range: 0–1)	0.14 (0.11)
Perceived neighborhood disorder (range: 1–7) ^a^	2.48 (1.41)
Age (in years)	65.73 (9.93)
Sex	
Men	46.52%
Women	53.48%
Race/Ethnicity ^b^	
Non-Hispanic white	76.69%
Non-Hispanic Black	10.02%
Hispanic	9.19%
Non-Hispanic other	4.10%
Marital status	
Married/Partnered	66.18%
Divorced/Separated	14.35%
Widowed	12.60%
Never married	6.86%
Education (in years)	13.36 (2.92)
Total annual household income ($)	89792.1 (134551.6)
Activities of daily living (ADL)	
No limitations	85.29%
One or more limitations	14.71%

^a^ Greater values indicate greater sense of hopelessness and greater perceived neighborhood disorder.

^b^ Racial/ethnic identification of ‘non-Hispanic other’ indicated American Indian, Alaskan Native, Asian, and Pacific Islander.

*Note*. All estimates were weighted.

Table 2 presents the results of the linear regression models predicting hopelessness. In Model 1, a significant association was observed between residing in neighborhoods with higher poverty levels and increased feelings of hopelessness (b = 0.11, 95% CI = 0.08, 0.15, *p* < .001), after adjusting for individual sociodemographic and health factors. Similarly, in Model 2, we found that higher levels of perceived disorder were associated with a greater sense of hopelessness (b = 0.16, 95% CI: 0.14, 0.19, *p* < .001). In Model 3 where both poverty and disorder were included, the association between neighborhood poverty and hopelessness weakened substantially; the magnitude of the coefficient was reduced by nearly two thirds (b = 0.04, 95% CI = 0.0003, 0.07, *p* = .048). However, the association of perceived neighborhood disorder with hopelessness remained robust, identical to the magnitude observed in Model 2 (b = 0.16, 95% CI = 0.14, 0.18, *p* < .001).

**Table 2:** Linear regression models estimating hopelessness, 2014/2016 health and retirement study, age 50+ (N = 12,284).

	Model 1		Model 2		Model 3	
	B	95% CI	B	95% CI	B	95% CI
***Main Predictors***						
Neighborhood poverty (logged)	0.11***	(0.08,0.15)			0.04*	(0.0003,0.07)
Perceived neighborhood disorder			0.16***	(0.14,0.19)	0.16***	(0.14,0.18)
***Confounders***						
Age (in years)	0.001	(-0.002,0.004)	0.002	(-0.002,0.01)	0.002	(-0.002,0.01)
Women	-0.10***	(-0.15,-0.05)	-0.10***	(-0.16,-0.05)	-0.10***	(-0.15,-0.05)
Race/ethnicity						
(Ref: non-Hispanic white)						
Non-Hispanic Black	-0.22***	(-0.31,-0.13)	-0.28***	(-0.37,-0.19)	-0.30***	(-0.39,-0.21)
Hispanic	-0.09	(-0.21,0.02)	-0.10	(-0.21,0.01)	-0.12*	(-0.23,-0.00)
Non-Hispanic other	0.16*	(0.01,0.31)	0.14	(-0.01,0.29)	0.14	(-0.01,0.29)
Marital status						
(Ref: Married/partnered)						
Divorced/separated	0.06	(-0.03,0.15)	0.05	(-0.03,0.14)	0.05	(-0.03,0.14)
Widowed	0.01	(-0.08,0.10)	0.03	(-0.06,0.12)	0.02	(-0.07,0.11)
Never married	0.10	(-0.03,0.23)	0.06	(-0.07,0.19)	0.06	(-0.08,0.19)
Years of education	-0.09***	(-0.10,-0.08)	-0.09***	(-0.10,-0.07)	-0.09***	(-0.10,-0.07)
Household income (logged)	-0.11***	(-0.14,-0.09)	-0.11***	(-0.13,-0.08)	-0.11***	(-0.13,-0.08)
Limitations in any ADLs	0.52***	(0.43,0.61)	0.48***	(0.39,0.57)	0.48***	(0.39,0.57)
Constant	4.88***	(4.45,5.31)	4.06***	(3.64,4.49)	4.12***	(3.69,4.54)

*p < .05;

**p < .01;

***p < .001.

^a^ Activities of daily living.

We further examined the formal mediating effects of neighborhood disorder using the SEM framework. First, the confirmatory factor analysis (CFA) for the measurement model was conducted. This model included two latent factors–perceived neighborhood disorder and hopelessness. Each was represented by four observed items. The model demonstrated good fit: RMSEA = 0.027 (90% CI = 0.023, 0.030), CFI = 0.991, and SRMR = 0.016. All factor loadings were high, (β = 0.65–0.82 for disorder) and (β = 0.71–0.86 for hopelessness), suggesting that the observed indicators adequately measured their respective latent constructs.

**Fig 1:**
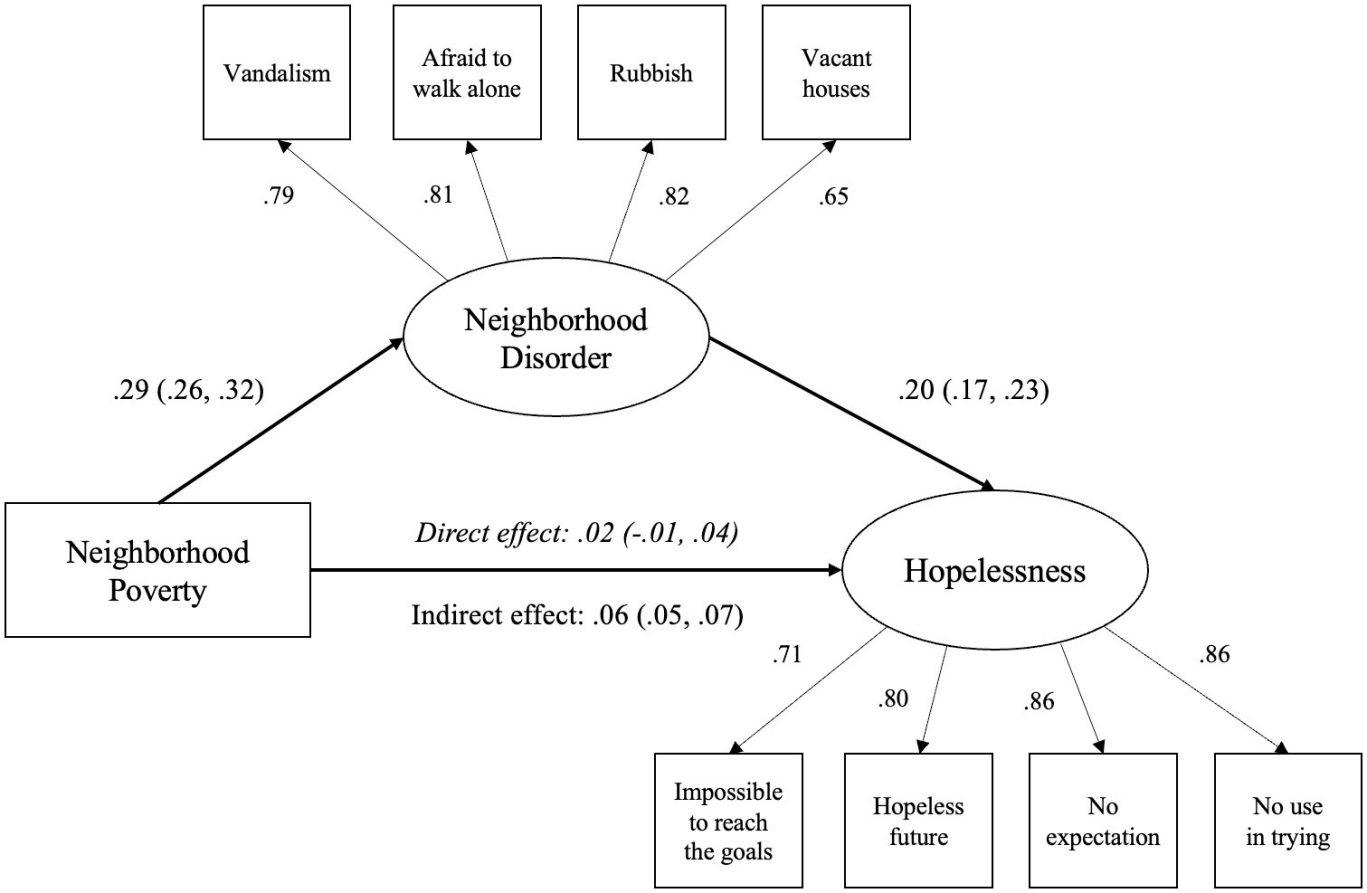
The mediation effect of neighborhood disorder. *Note*. Numbers indicate standardized regression coefficients with bootstrapped 95% confidence intervals in parentheses. The model adjusted for age, gender, race/ethnicity, marital status, education, household income, and any limitations in activities of daily living; Model fit: CFI = 0.975; SRMR = 0.027; RMSEA = 0.024 (90% CI = 0.022, 0.026).

In the structural model, we examined the direct and indirect pathways from neighborhood poverty to hopelessness, mediated by neighborhood disorder. The model fit was good: RMSEA = 0.021 (90% CI = 0.019, 0.023), CFI = 0.980, and SRMR = 0.017. The total effect of neighborhood poverty on hopelessness was significant (β = 0.08, bootstrapped 95% CI = 0.05, 0.10). Decomposition of effects, as shown in Fig 1, revealed that the direct effect of neighborhood poverty was not significant (β = 0.02, bootstrapped 95% CI = -0.01, 0.04), while the indirect effect through neighborhood disorder was significant (β = 0.06, bootstrapped 95% CI = 0.05, 0.07). The calculated effect size by proportion mediated approach was 0.75, indicating that neighborhood disorder mediated 75% of the association between neighborhood poverty and hopelessness.

We conducted several sensitivity analyses to validate the robustness of our findings. First, we examined the potential for nonlinearities in the associations by replicating the analyses using binary indicators to define high neighborhood poverty (10% or higher) and high disorder (scores above the median’s top half). The results, shown in S1 Table and S1 Fig, demonstrate that high neighborhood poverty was significantly associated with higher levels of hopelessness, both directly and indirectly through high neighborhood disorder, with the indirect effects of disorder accounting for 43% of the total effect of poverty. Second, to account for differences in propensity towards reporting hopeless, we adjusted for respondents’ prior levels of hopelessness measured in the 2010/2012 HRS SAQ (N = 9,719). As shown in the S2 Table, the association between neighborhood poverty and hopelessness remained even after controlling for initial levels of hopelessness. However, this association became null after neighborhood disorder was added to the model. The SEM analysis, as shown in S2 Fig, indicated that neighborhood disorder entirely mediates the relationship between neighborhood poverty and hopelessness, with no direct effect of poverty observed.

## Discussion

Hopelessness has been identified as a robust predictor of health, well-being, and mortality, particularly for older populations, because maintaining a positive outlook for the future is a key source of motivation and adaptation [53–55]. Previous research predominantly focused on individual-level socioeconomic conditions to predict hopelessness. However, this study, grounded in hopelessness theory [13], which proposes that uncontrollable and adverse environmental conditions can induce a sense of hopelessness, provides the first empirical evidence that neighborhood poverty plays a significant role as an environmental factor shaping hopelessness utilizing a nationally representative sample of older Americans. As anticipated, residents of more impoverished neighborhoods were more likely to report a greater sense of hopelessness, adjusting for individual level sociodemographic and health factors. This aligns with previous research that reported an association between neighborhood poverty and hopelessness in young adults [23]. Moreover, our finding is consistent with prior studies reporting significant associations between neighborhood-level unemployment, another indicator of neighborhood socioeconomic disadvantage, and hopelessness. Specifically, drawing data from a population-representative survey of adults aged 20 and older in Chicago, IL, Mair and colleagues [56] found that higher neighborhood unemployment rates, defined based on the US Census data, predicted increased odds of reporting hopelessness. In a study of Swiss adults aged 40 and older, Morselli [46] reported that individuals living in cantons (the federal states) with high unemployment rates were more likely to feel hopeless. Along with the collective evidence, our finding confirms and underscores the importance of neighborhood socioeconomic factors in shaping residents’ sense of hopelessness, above and beyond personal socioeconomic status.

Furthermore, neighborhood disorder explained the association between neighborhood poverty and hopelessness. To the best of our knowledge, this study is the first that examined the underlying process of neighborhood poverty and hopelessness. This finding aligns with previous studies reporting a crucial role of neighborhood disorder linking neighborhood SES and health [27, 39]. The social disorganization theory provides a theoretical basis to interpret the observed mediating process of poverty through perceived disorder [25]. This theory proposes that social and structural factors within impoverished neighborhoods, such as social instability, weakened social institutions, and the lack of resources, create conditions that foster disorder within a community. Empirical evidence from prior research reporting relatively higher levels of neighborhood disorder within socioeconomically disadvantaged neighborhoods further supports our finding [25, 29–31, 57]. The visible signs of decay and a lack of social control within impoverished neighborhoods can impact residents’ perceptions of personal safety [44]. Additionally, this ongoing exposure may lead residents feel that they do not have control over their environments [58, 59], intensifying feelings of hopelessness, a negative expectation for future improvement or success.

Findings of this study have potentially important implications for intervention strategies. Given the association between neighborhood poverty and hopelessness, one potential approach could involve providing subsidies to low-income older adults to improve their housing options. For example, the Moving to Opportunity program, which provided housing vouchers to move families from high- to low-poverty neighborhoods, has shown improved mental health outcomes, including reduced psychological distress and depression, though this was a study of children and their parents rather than older adults [60]. Another potential approach is focusing on reducing neighborhood poverty more broadly. This could include adopting comprehensive social policies that address the root causes of poverty by improving access to education, healthcare, and employment opportunities within impoverished neighborhoods [61]. Community investment programs, such as the New Market Tax Credits, that aim to revitalize marginalized, low-income neighborhoods by supporting housing rehabilitation, economic development, and infrastructure improvements have also proven effective in improving educational outcomes, employment rates, and household income, ultimately reducing neighborhood poverty [62, 63].

While addressing structural factors necessitates a fundamental restructuring of society, which is challenging, our findings highlight the critical role of neighborhood disorder. This recognition opens the avenue for more attainable interventions aimed at mitigating despair in impoverished neighborhoods by disrupting the link between poverty and disorder. Community intervention strategies, therefore, could focus on creating safer, more orderly neighborhoods. This might involve initiatives or regulations aimed at sustained reductions in local disorder and improving the overall quality of life in socioeconomically disadvantaged areas. Graffiti prevention and removal program is one of the most common initiatives that local governments have. The City of Philadelphia enforced a Doors and Windows Ordinance that required property owners of abandoned buildings to install working doors and windows. Community-oriented, problem-solving policing (e.g., door-to-door visits, homeless removal) to reduce social and physical disorder in high-risk neighborhoods have been also found to be effective in reducing residents’ perceptions of social and physical disorder and increasing their feelings of safety [64, 65].

### Limitation

This study has several limitations worth noting for interpretations of the findings. First, our use of cross-sectional data precludes the possibility of drawing causal inferences. Although we conducted additional analyses adjusting for baseline levels of hopelessness to minimize the potential reverse causality issues, the establishment of causal associations remains beyond the scope of this study. Second, while we controlled for key individual-level covariates, the potential for bias arising from residual confounding cannot be entirely ruled out. Lastly, our use of subjective measures to assess neighborhood disorder and hopelessness might have introduced bias related to individual variations in perception.

## Conclusions

While previous studies on psychological well-being mostly focused on individual characteristics, a growing body of literature emphasizes that neighborhood context matters for individual psychological well-being. We provided the first population-level findings that neighborhood poverty was associated with a sense of hopelessness among older adults, and the association was explained by neighborhood disorder. Our results suggest that promoting psychological well-being calls for attention to both individual and contextual factors. In addition to neighborhood disorder, further research is needed to investigate other modifiable factors that link neighborhood poverty to poor psychosocial well-being outcomes.

## Supporting information

S1 Table Sensitivity analysis with binary indicator (N = 12,284). (DOCX)

S2 Table Sensitivity analysis with prior levels of hopelessness (N = 9,719). (DOCX)

S1 Fig The mediation effect of perceived neighborhood disorder with binary indicators. (DOCX)

S2 Fig The mediation effect of perceived neighborhood disorder adjusting for prior hopelessness. (DOCX)
