# Supplementary material for: Neighborhood poverty and hopelessness in older adults: The mediating role of perceived neighborhood disorder
Source: PLoS One. 2024 Oct 15;19(10):e0311894. doi: 10.1371/journal.pone.0311894 (PMC11478814; doi:10.1371/journal.pone.0311894)
Supplement: S1 Table — (DOCX) [file pone.0311894.s001.docx]

**S1 Table.** **Sensitivity Analysis with Binary Indicator (N=12,284).**

|  | Model 1 | | Model 2 | | Model 3 | |
| --- | --- | --- | --- | --- | --- | --- |
|  | B | 95% CI | B | 95% CI | B | 95% CI |
| High neighborhood poverty | 0.20 *** | (0.14,0.26) |  |  | 0.11 *** | (0.06,0.17) |
| High perceived disorder |  |  | 0.42 *** | (0.36,0.48) | 0.39 *** | (0.34,0.45) |

****p*<.001.

All models controlled for individual-level sociodemographic and health factors. High neighborhood poverty was defined as 10% or higher. High perceived disorder was defined as a top half of the median.
