# Supplementary material for: Neighborhood poverty and hopelessness in older adults: The mediating role of perceived neighborhood disorder
Source: PLoS One. 2024 Oct 15;19(10):e0311894. doi: 10.1371/journal.pone.0311894 (PMC11478814; doi:10.1371/journal.pone.0311894)
Supplement: S2 Table — (DOCX) [file pone.0311894.s002.docx]

**S2 Table. Sensitivity Analysis with Prior Levels of Hopelessness (N=9,719).**

|  | Model 1 | | Model 2 | | Model 3 | |
| --- | --- | --- | --- | --- | --- | --- |
|  | B | 95% CI | B | 95% CI | B | 95% CI |
| Prior Levels of Hopelessness | 0.51 *** | (0.48,0.54) | 0.50 *** | (0.47,0.53) | 0.50 *** | (0.47,0.53) |
| Neighborhood poverty (logged) | 0.04 ** | (0.01,0.07) |  |  | 0.00 | (-0.03,0.03) |
| Perceived disorder |  |  | 0.10 *** | (0.08,0.12) | 0.10 *** | (0.08,0.12) |

***p*<.01; ****p*<.001.

Prior levels of hopelessness were measured 4 years ago (2010/2012). All models controlled for individual-level sociodemographic and health factors.
