# Supplementary material for: Neighborhood poverty and hopelessness in older adults: The mediating role of perceived neighborhood disorder
Source: PLoS One. 2024 Oct 15;19(10):e0311894. doi: 10.1371/journal.pone.0311894 (PMC11478814; doi:10.1371/journal.pone.0311894)
Supplement: S1 Fig — (DOCX) [file pone.0311894.s003.docx]

**S1 Fig.** **The Mediation Effect of Perceived Neighborhood Disorder with Binary Indicators.**

*Note.* Numbers indicate standardized regression coefficients with bootstrapped 95% confidence intervals in parentheses. The model adjusted for age, gender, race/ethnicity, marital status, education, household income, and any limitations in activities of daily living.
