# Supplementary material for: Neighborhood poverty and hopelessness in older adults: The mediating role of perceived neighborhood disorder
Source: PLoS One. 2024 Oct 15;19(10):e0311894. doi: 10.1371/journal.pone.0311894 (PMC11478814; doi:10.1371/journal.pone.0311894)
Supplement: S2 Fig — (DOCX) [file pone.0311894.s004.docx]

**S2 Fig.** **The Mediation Effect of Perceived Neighborhood Disorder Adjusting for Prior Hopelessness.**

*Note.* Numbers indicate standardized regression coefficients with bootstrapped 95% confidence intervals in parentheses. The model adjusted for age, gender, race/ethnicity, marital status, education, household income, any limitations in activities of daily living, and prior levels of hopelessness.
